# Primary One Stage Reconstruction in Complex Facial Avulsion Injury

**Published:** 2017-09

**Authors:** Abhishek Ghosh

**Affiliations:** Poona Hospital, Noble Hospital, Pune, India

**Keywords:** Hemiface, Orbit, Zygoma, Avulsion, Degloving

## Abstract

Complex facial injuries with soft tissue degloving and bony avulsion are very devastating to the patient. Partial degloving injuries are described but hemifacial degloving with zygoma avulsion are rare. The author presents a case of post-traumatic degloving of the left upper lip, nose, part of forehead, upper and lower eyelids and cheek with avulsion of the left zygoma. The management included immediate resuscitation and early surgery to reposition the skeletal as well as soft tissue avulsion. The wound was thoroughly washed and primary repositioning and fixation were done. Early one stage surgery with meticulous debridement and alignment of the anatomical landmarks results in very good aesthetic and functional outcome.

## INTRODUCTION

The face is a very important part of the human body giving identity and sense of confidence to a person. Any post traumatic facial deformity not only causes functional problems but also psychosocial dysfunction. This article reports a case of post-traumatic degloving of majority of left side of the face with avulsion of the zygomatic bone. There are various reports suggesting better outcomes with early primary reconstruction with less risk of infection^1^^-^^3^ and also better aesthetic outcome.^4^^,^^5^ An early single staged surgical correction was done for this case which gave very good functional and aesthetic outcome. 

## CASE REPORT

**Fig. 1:**
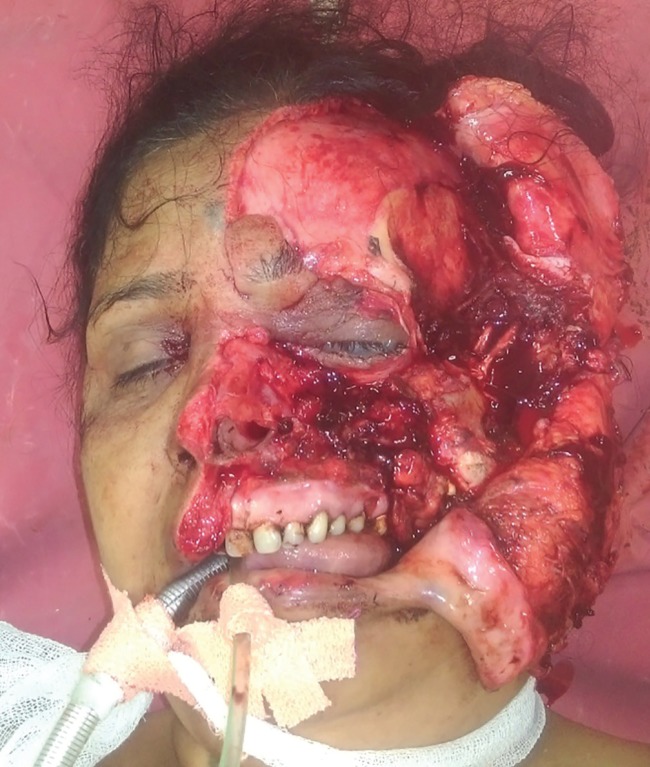
Preoperative picture showing complete hemifacial and zygomatic avulsion

A 56 years old female presented with history of road traffic accident with resultant avulsion of her left side of face. There was degloving and avulsion of the left hemi-face including the upper lip nose, forehead skin and eyebrow, upper and lower eyelids and the entire left cheek. The zygomatic bone was also avulsed and displaced laterally. The globe was displaced inferiorly towards the maxillary sinus (Figure 1).

The patient was immediately resuscitated airway management, control of bleeding and maintainence of circulation. Other injuries were quickly ruled out and CT scan with 3D CT of the face was done. CT scan showed avulsion of the zygoma with lateral blowout fracture of the orbit. There was no brain injury. The vision was tested for finger counting which was present at 2 to 3 feet. Decision was taken for an immediate single stage reconstruction of the bony as well as soft tissue avulsion.

**Fig. 2:**
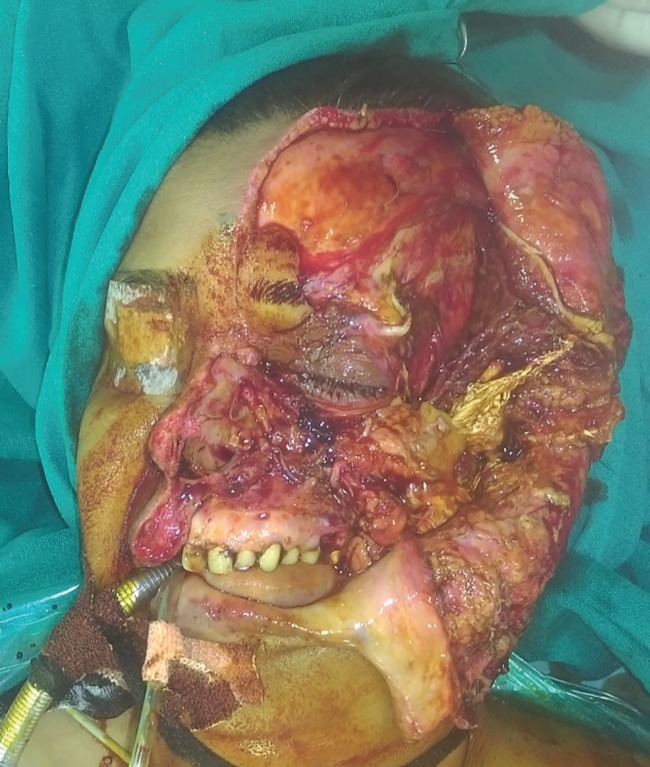
Preoperative picture after debridement and wash

**Fig. 3:**
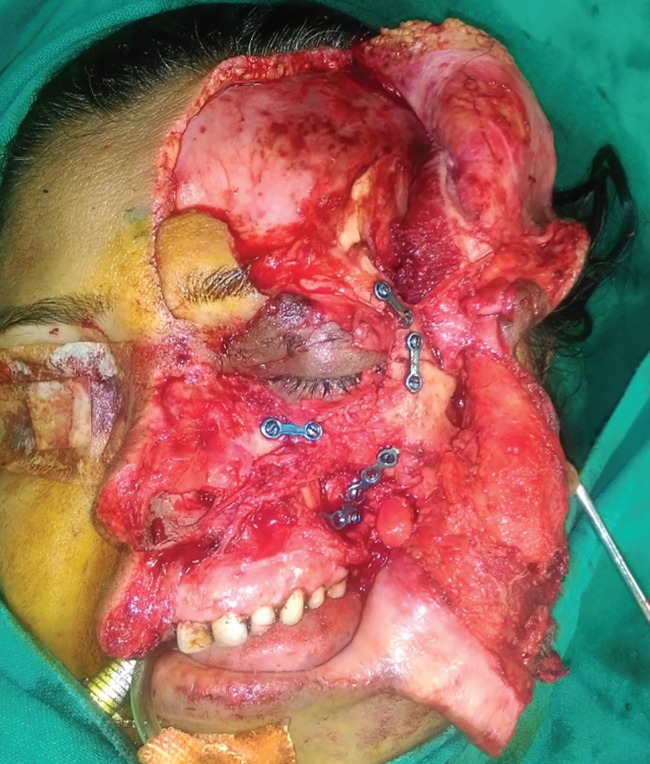
Orbit reformed with pieces of zygomatic bone brought into position and fixed with titanium implants

The patient was taken for surgery and under anesthesia the wound was thoroughly washed with normal saline and diluted betadine solution. All contaminants and foreign bodies were removed. Careful debridement was done and crushed nonviable tissues were excised. The orbital cavity was then reconstructed. The globe was seen to be displaced towards the maxillary sinus. The avulsed zygoma still had soft tissue attachments. There was a piece of lateral orbital rim with the soft tissue flap which was repositioned and the lateral orbital wall was reformed. The fractures were fixed with titanium miniplates. The eyeball was thus repositioned to its original position and the volume of the orbital cavity was restored (Figure 2 and 3).

**Fig. 4:**
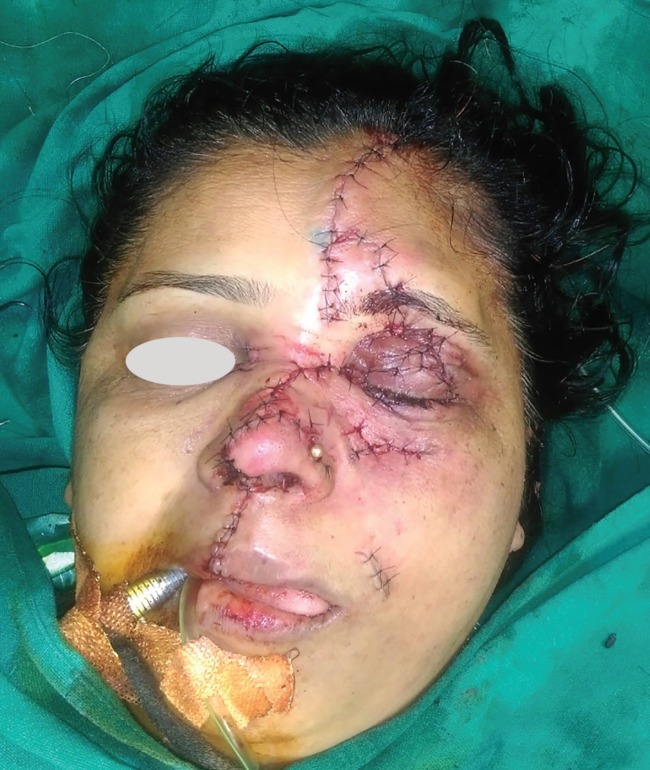
Postoperative results.

**Fig. 5:**
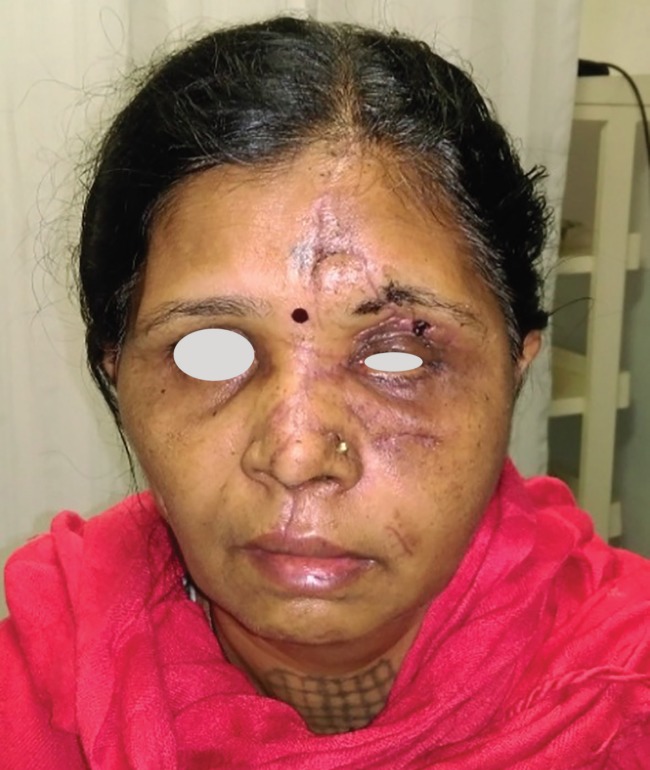
Postoperative results after 1 month

Once the bony fixation was done the soft tissue reconstruction was started. The degloved flap was repositioned. Tacking sutures were taken to the periosteum wherever possible to keep the flap in place. The upper lip was repaired in layers after marking the anatomical landmarks. The mucosa and muscle were repaired with absorbable sutures and skin with nylon. The nose was reconstructed with repair of the mucosal lining followed by repair of the cartilage framework. The nasal ala was repositioned and the skin was repaired. The eyelids and forehead were repaired in layers (Figure 4). The post-operative course was uneventful. The patient had no problems in visual acuity. The patient was on higher antibiotics for five days and discharged. The sutures were removed on 7^th^ postoperative day. All wounds healed well without any flap necrosis (Figure 5). The patient had normal vision and full facial functions at six months follow up.

## DISCUSSION

Complex hemifacial avulsion injuries are challenging and difficult to treat. The most common causes are high velocity trauma and assault. There is not only a soft tissue degloving but also a bony avulsion in such cases. The problem is compounded by the presence of foreign bodies and contaminations including dirt and stone particles. Early single stage reconstruction provides excellent functional and aesthetic outcomes. Thorough washing and removal of foreign bodies and precise debridement help to prevent infection and flap loss.^6^^-^^8^ Meticulous matching of the anatomical landmarks and repair in anatomical layers gives very good final results. Immediate soft tissue reconstruction results in less scarring and infection. Delayed repair results in oedema developing which obscures the anatomical landmarks and give inferior aesthetic results. There is higher risk of infection too in case of delayed repair. The complete avulsion with lateral displacement of the zygoma is a rare occurrence and it causes displacement of the globe inferiorly and outwards. Early repositioning of the bony fragments and rigid orbit reconstruction repositions the globe and helps maintain visual acuity.^9^^-^^11^

Surgical management of unusual complex problems is highly challenging as they do not follow any set protocols. Sticking to the basic tenets of reconstruction with matching of the anatomical landmarks and reconstructing in layers gives very satisfying outcomes even in the most ghastly injuries. Immediate single stage procedure with meticulous reconstruction is the key to excellent functional and aesthetic results.

## CONFLICT OF INTEREST

The authors declare no conflict of interest.
